# Bumblebees can discriminate between scent-marks deposited by conspecifics

**DOI:** 10.1038/srep43872

**Published:** 2017-03-07

**Authors:** Richard F. Pearce, Luca Giuggioli, Sean A. Rands

**Affiliations:** 1 School of Biological Sciences, University of Bristol, Bristol, UK; 2 Bristol Centre for Complexity Sciences, University of Bristol, Bristol, UK; 3 Department of Engineering Mathematics, University of Bristol, UK

## Abstract

Bumblebees secrete a substance from their tarsi wherever they land, which can be detected by conspecifics. These secretions are referred to as scent-marks, which bumblebees are able to use as social cues. Although it has been found that bumblebees can detect and associate scent-marks with rewarding or unrewarding flowers, their ability at discriminating between scent-marks from bumblebees of differing relatedness is unknown. We performed three separate experiments with bumblebees (*Bombus terrestris*), where they were repeatedly exposed to rewarding and unrewarding artificial flowers simultaneously. Each flower type carried scent-marks from conspecifics of differing relatedness or were unmarked. We found that bumblebees are able to distinguish between 1. Unmarked flowers and flowers that they themselves had scent-marked, 2. Flowers scent-marked by themselves and flowers scent-marked by others in their nest (nestmates), and 3. Flowers scent-marked by their nestmates and flowers scent-marked by non-nestmates. The bumblebees found it more difficult to discriminate between each of the flower types when both flower types were scent-marked. Our findings show that bumblebees have the ability to discriminate between scent-marks of conspecifics, which are potentially very similar in their chemical composition, and they can use this ability to improve their foraging success.

Bumblebees secrete a substance from their tarsus consisting of a mixture of hydrocarbons^1^,^2^, which primarily helps them adhere to surfaces (such as flowers) and reduces desiccation^1^,^3^,^4^,^5^. The secretion of these cuticular hydrocarbons (CHCs) is not exclusive to bumblebees, but also occurs in wasps, ants, termites, and honeybees, and can play a role in nestmate recognition^6^,^7^,^8^. In social insects, these CHCs may be different between individuals of a different species (heterospecifics), nest (non-nestmates), or between individuals of the same nest (nestmates). The composition of each individual’s CHCs can be referred to as their signature mixture which may be used by other individuals to aid nestmate recognition^9^.

There are many ways in which bumblebees are able to use chemical signals and cues in the context of intraspecific and interspecific communication^10^. Bumblebees (*Bombus occidentalis*) are able to discriminate between nest entrances that are contaminated with their own nest’s odour from nest entrances contaminated with heterospecifics or foreign conspecifics^11^. More specifically, bumblebees (*B. terrestris*) have been found to be able to discriminate between the wax scents from a foreign colony and their own colony, suggesting that this may allow workers to recognise their own colony^12^. Bumblebees may also be able to use nestmate recognition *via* individually-borne cues as a way of avoiding inbreeding^13^,^14^. Social parasitic cuckoo bumblebees (subgenus *Psithyrus*) show a preference for, and therefore are able to recognise, the volatile signals of their host species^15^. Furthermore, cuckoo bumblebees have the ability to mimic specific CHCs of their bumblebee hosts, enabling them to invade their hosts^16^. CHCs are deposited onto any surface the bumblebee lands on, such as their nest entrance and flowers^3^,^17^. These deposits can remain for over 24 hours^18^ and have a scent that can be detected by bumblebees^19^, and therefore are referred to as scent-marks.

Foraging Bumblebees can use scent-marks left behind on flowers visited by themselves, conspecifics and, in some cases, heterospecifics as social cues that may indicate the level of reward present in the flower^10^,^20^,^21^,^22^,^23^,^24^. This is an example of associative learning. Scent-marks left by bumblebees at food, neutral, or nest sites are indistinguishable in terms of their chemical composition^17^. Bumblebees also respond similarly towards flowers that contain scent-marks originally deposited at the nest entrance and scent-marks deposited on recently visited flowers^25^. Furthermore, the chemical composition (hydrocarbons) of bumblebee scent-marks were almost identical to that found on the epicuticular region of their tarsus^25^. This all indicates that bumblebees are inadvertently depositing general chemical footprints wherever they walk that act as cues and not signals.

These scent-marks can act as attractants or repellents (or neither) depending on the bumblebee’s experience with the level of reward found in the flower and how easily it is obtained^26^,^27^,^28^. Although naïve (inexperienced) bumblebee foragers have no innate avoidance or preference for scent-marked flowers^29^, there is evidence that bumblebees can use scent-marks as an indication that a flower either contains nectar^19^,^30^,^31^ or, as is more commonly seen in the wild, that a flower has had its nectar depleted^20^,^32^,^33^,^34^,^35^. There are no existing studies to show that bumblebees can discriminate or have an innate preference between their own scent-mark and the scent-marks of nestmates, or between scent-marks of nestmates and scent-marks of non-nestmates.

In the wild it would be beneficial for bumblebees to detect their own scent-mark, as they could use this to inform them of depleted flowers they have visited recently or of rewarding flowers they have visited a sufficient length of time ago due to changes in the scent-mark’s chemical composition over time^32^,^33^, thus selecting more rewarding flowers over less rewarding ones. The bee would also not need to rely solely on spatial memory to identify where it has already foraged. However, these benefits of scent marking may not always hold true if there are greater time or energy costs associated with scent marking or detection than there are benefits. It remains to be seen whether bumblebees are able to use just their own personal scent-mark to detect the absence of a floral reward.

Past laboratory experiments have used scent-marks from different individuals to the ones being tested (either from the same or a different nest), by allowing mark-depositing bees to visit the flowers before the test bee^33^, or by experimentally adding tarsal extracts directly on to the flowers^27^. Although it has been investigated whether bumblebees can use their own scent-mark to gain information about floral nectar in the field^20^, this study is unable to confirm that bumblebees reject flowers purely because they contain their own scent-mark. This is because it cannot be known with certainty that only the test bee had landed on the flower. Furthermore, the flowers may have had their appearance altered once they had been visited, given that real flowers were used. Under laboratory conditions, it has been shown that bumblebees perform better than expected if foraging at random on an array of artificial flowers^27^, which were moved after each approach to account for spatial learning. This was concluded to be due to the avoidance of visited flowers *via* scent-mark detection. As part of our study, we intend to make this more explicit and also to take it further by assessing the bumblebee’s ability at discriminating between scent-marks from bumblebees of varying degrees of relatedness.

Bumblebees are likely to benefit from being able to discriminate between their own scent-mark and scent-marks from bumblebees within their nest (nestmates). Being able to do this would allow them to decide whether or not to forage on flowers they or their nestmates have visited in the past (personal vs social information), which would be particularly useful given that they use social information to complement personal experience^36^,^37^. It may also convey the degree of foraging area overlap the individual has with its nestmates, and possibly the size of the area covered by the colony, informing the forager where to forage. For these reasons, being able to discriminate between their own scent-mark and scent-marks from nestmates may increase a forager’s rate of nectar uptake.

Furthermore, being able to distinguish between scent-marks from nestmates and non-nestmates is also likely to be beneficial. It would allow individuals to decide whether or not to forage on flowers their nestmates or non-nestmates have visited in the past (social information), which could be useful if these two groups of foragers were selecting flowers of different quality. Perhaps more importantly, it would also convey the degree of foraging area overlap their nestmates may have with potential competitors’, thus informing them of the level of competition that they have for resources. High competition may result in a colony foraging elsewhere, possibly due to reduced flower constancy at high conspecific densities, which may then go on to affect the efficiency of pollination visits^38^. Conversely, the presence of conspecifics at low densities can initially provide beneficial information^38^,^39^.

We performed three different experiments to assess a bumblebee’s ability to distinguish between scent-marks deposited by bumblebees of varying degrees of relatedness. First we examined whether foraging bumblebees can learn to avoid unrewarding flowers scent-marked by themselves, while having a preference for rewarding flowers that are unmarked (Experiment 1). Second we examined whether foraging bumblebees can learn to avoid unrewarding flowers scent-marked by themselves while having a preference for rewarding flowers scent-marked by their nestmates (Experiment 2). Third we examined whether foraging bumblebees can learn to avoid unrewarding flowers scent-marked by non-nestmates while having a preference for rewarding flowers scent-marked by their nestmates (Experiment 3). Finally we compared the three experiments with each other.

## Results

**Figure 1:**
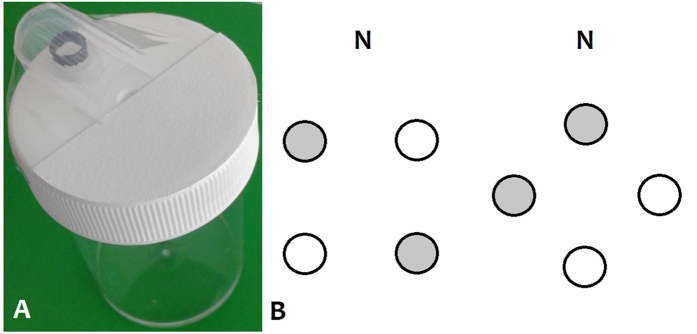
Flower structure and arrangements. (**A**) Artificial flower design, showing the well for storing sucrose or water, the translucent cover attached using transparent tape, and the semi-circle of filter paper at the entrance; (**B**) the two arrangements of four flowers, with rewarding (filled circles) and unrewarding (unfilled circles) flowers positioned randomly for each bout. The direction of the nest is indicated by **N**.

For each experiment, an individual foraging bumble was exposed to four artificial flowers (Fig. 1). Two flowers were rewarding (sucrose) and contained a given scent-mark, and two flowers were unrewarding (water) and contained a different scent-mark or no scent-mark, depending in the experiment (Table 1), thus creating two different flower types. The type and occurrence of the bumblebees’ initial interaction towards each of the four flowers was recorded during three testing phases, where all flowers contained water to control for the possibility of remote sucrose detection by the bees. Forms of interaction with each flower are defined as follows, with their notation in brackets: the bee hovers facing the flower but does not land (Hover), lands on the flower (Land), enters the flower but does not touch the well with its proboscis or antennae (Enter), and drinks from the well or extends proboscis (Drink). To succeed at this task, a bumblebee must be able to detect and sense a difference between the two scent-marks (scent-mark discrimination), but also be able to perform operant learning enabling it to associate one of the scent-marks with a reward and/or the other scent-mark with the absence of a reward (associative learning).

### Experiment 1: Own scent-mark vs No scent-mark

**Figure 2:**
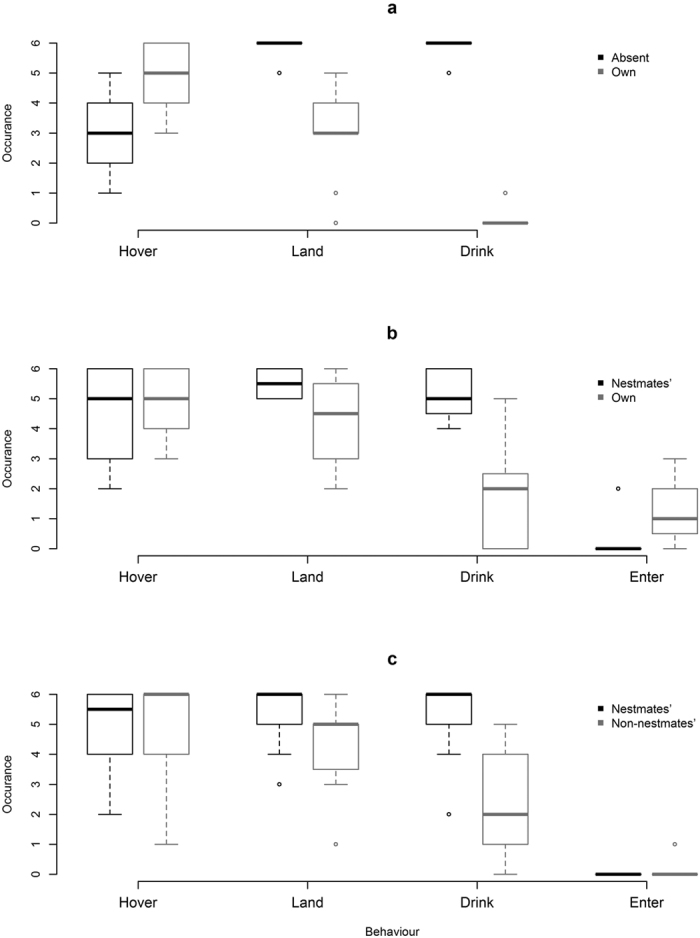
Number of occurrences of hovering (hovers facing the flower but does not land), landing (lands on the flower), drinking (attempts to drink), and entering (enters but does not attempt to drink) behaviour towards the six flowers of each flower type for the twelve bumblebees tested in each experiment. (**a)** Experiment 1: own scent-mark vs no scent-mark; (**b)** Experiment 2: own scent-mark vs nestmates’ scent-mark; (**c)** Experiment 3: non-nestmates’ scent-mark vs nestmates’ scent-mark.

The bees behaved differently towards the two flower types (Fig. 2a), with more hovering occurrences (pairwise Mann-Whitney-Wilcoxon test: n = 12, U = 55, p = 0.0055), but fewer landings (n = 12, U = 0, p = 0.0022) and drinking occurrences (n = 12, U = 0, p = 0.0015) on scent-marked flowers. There were no occurrences of a bee entering the flower without touching the well with its proboscis or antennae (Enter). There was only one instance of a bee not interacting with a flower, which occurred for a scent-marked flower.

**Figure 3:**
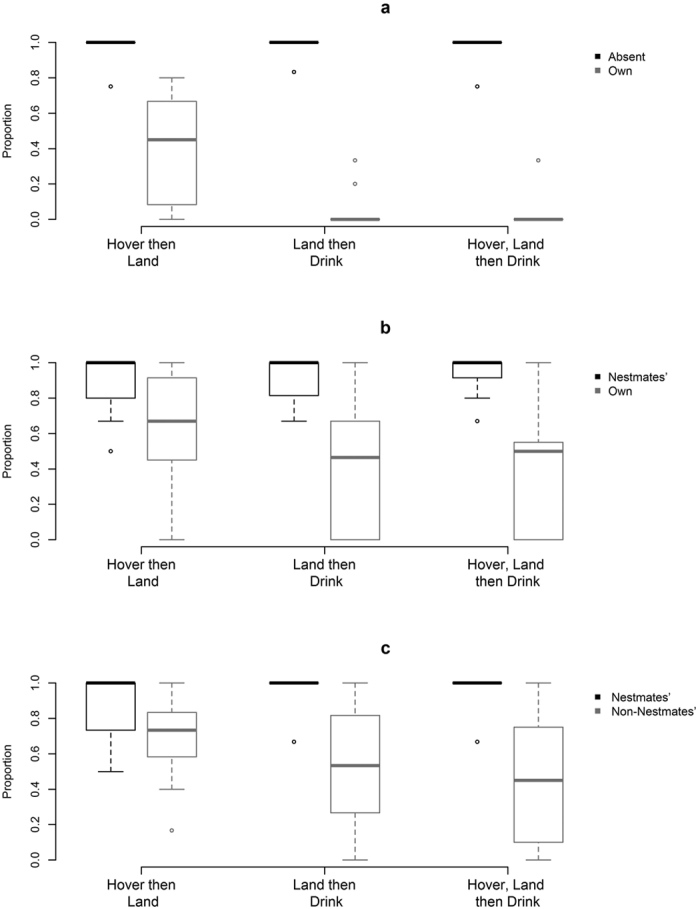
Dependent behaviour as a proportion of the initial behaviour for the twelve bumblebees tested in each experiment. (**a**) Experiment 1: own scent-mark vs no scent-mark; (**b)** Experiment 2: own scent-mark vs nestmates’ scent-mark (only eleven bees qualified for the hover, land then drink dependent behaviour); and (**c)** Experiment 3: non-nestmates’ scent-mark vs nestmates’ scent-mark. Behaviours include hovering (hovers facing the flower but does not land), landing (lands on the flower), and drinking (attempts to drink).

The bee’s initial behaviour affected its next decision (Fig. 3a). For the scent-marked flowers, bees that hovered were less likely to land on the flower (n = 12, U = 0, p = 0.0038), bees that landed were less likely to drink (n = 12, U = 0, p = 0.0012), and bees that hovered then landed were less likely to drink (n = 12, U = 0, p = 0.0009), when compared to unmarked flowers.

The mean duration of the initial physical interaction with each of the flowers (the time from landing to departing) was calculated for the final five bees tested. They spent more time on the unmarked flowers than the scent-marked flowers (*t*_4_ = 5.3318, p = 0.0060), with a mean time of 10.70 ± 4.40 and 0.72 ± 0.54 seconds respectively.

### Experiment 2: Own scent-mark vs Nestmate’s scent-mark

The bees behaved differently towards the two flower types (Fig. 2b), landing (n = 12, U = 4, p = 0.0308) and drinking (n = 12, U = 0, p = 0.0037) more frequently on flowers scent-marked by nestmates. Entering the flower without touching the well with its proboscis or antennae (Enter) was much more common for the flowers containing the bee’s own scent-mark (n = 12, U = 42, p = 0.0217). There was no difference between the two flower types in terms of hovering (n = 12, U = 15, p = 0.3869) and there were no occurrences of bees not interacting with a flower.

For the own-scent-marked flowers, bees that hovered were less likely to land on the flower (n = 12, U = 7.5, p = 0.0258), bees that landed were less likely to drink (n = 12, U = 0, p = 0.0038), and bees that hovered then landed were less likely to drink (n = 11, U = 0, p = 0.0058), when compared to flowers scent-marked by nestmates (Fig. 3b).

Although the amount of data obtained was too small (n = 3), we observed that the bees spent more time on flowers scent-marked by nestmates than flowers scent-marked by themselves.

### Experiment 3: Non-nestmate’s scent-mark vs Nestmate’s scent-mark

Bees behaved differently towards the two flower types (Fig. 2c), landing (n = 12, U = 2.5, p = 0.0330) and drinking (n = 12, U = 1.5, p = 0.0055) more frequently on flowers scent-marked by nestmates. Entering the flower without touching the well with its proboscis or antennae (Enter) occurred only twice, each time by a bee interacting with a flower scent-marked by non-nestmates. There was no apparent difference between the two flower types in terms of hovering (n = 12, U = 12, p = 0.7768) and there were no occurrences of any bee not interacting with a flower.

For the flowers scent-marked by non-nestmates, bees that hovered were less likely to land on the flower (n = 12, U = 4, p = 0.0322), bees that landed were less likely to drink (n = 12, U = 1, p = 0.0080), and bees that hovered then landed were less likely to drink (n = 12, U = 1, p = 0.0078), when compared to the flowers scent-marked by nestmates (Fig. 3b).

Although the amount of data obtained was too small (n = 2) for analysis, we observed that the bees spent more time on flowers scent-marked by nestmates than flowers scent-marked by non-nestmates.

### Learning speed

**Figure 4:**
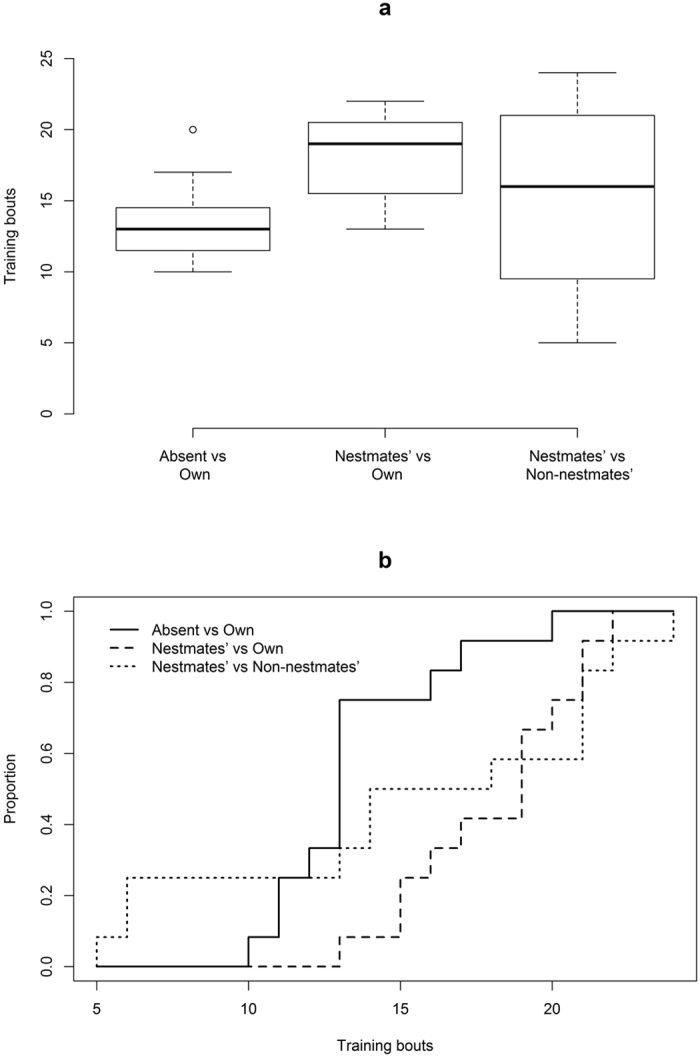
The learning speed of the bumblebees for each experiment. (**a)** The number of training bouts required before progressing to the testing phase; (**b)** the cumulative distribution of the proportion of bees that progressed to the testing phase given the number of training bouts. Experiment 3 is included for comparison, but is not directly relatable to Experiments 1 and 2 due to slightly different methods of obtaining scent-marks.

For Experiment 1 a median of 13 training bouts were required before moving on to the testing phase, with a range of 10 (Fig. 4a). For Experiment 2 the median was 19 with a range of 9, with its discrete cumulative distribution shifted to the right of that of Experiment 1 (Fig. 4b), showing that the bees required more training bouts before moving onto the testing phase (two-sample Kolmogorov-Smirnov test: D = 0.6667, p = 0.0097). For Experiment 3 the number of training bouts required by each of the twelve bees before moving onto the testing phase was highly variable (Fig. 4a), with a median of 16 and a range of 19. Some of the bees managed to learn by the sixth training bout, but twenty-four training bouts were required until all the bees had progressed to the testing phase (Fig. 4b).

## Discussion

Not only can bumblebees detect their own scent-mark, they can also discriminate between that and scent-marks of their nestmates, as well as between scent-marks of their nestmates and scent-marks of non-nestmates.

The bees were able to associate their own scent-mark with the absence of reward when these flowers were alongside rewarding flowers scent-marked by their nestmates (Experiment 2), but especially when alongside rewarding unmarked flowers (Experiment 1). The bees were also able to associate flowers that were scent-marked by non-nestmates with the absence of reward when alongside rewarding flowers that were scent-marked by their nestmates (Experiment 3). This ability to discriminate between flower types was evident by their avoidance of unrewarding flowers and their attraction towards rewarding flowers that emerged after repeat exposure to the two flower types simultaneously (Fig. 2).

There was no difference in hovering behaviour between the two flower types for both Experiments 2 and 3, but there was for Experiment 1. In Experiment 1 they almost always landed and drank from rewarding flowers, and almost always avoided drinking on unrewarding flowers. This was not true for Experiments 2 and 3, where they weren’t as likely to land or drink on rewarding flowers, and were more likely to drink from unrewarding flowers, when compared to Experiment 1. These behaviours suggest that the bees found it harder to discriminate between the two flower types when both were scent-marked. It has been found that it can sometimes be advantageous for bumblebees to make quick, inaccurate decisions^40^, but as this was not observed in Experiment 1 it is unlikely to be the reason for what was observed in Experiments 2 and 3.

The dependent behaviours observed in each of the experiments (Fig. 3) showed that hovering around an unrewarding flower was sometimes not enough to reject it by not landing. By landing on these flowers the bees were more likely to reject the flower by not drinking from them. Although bumblebees can detect odours remotely using their antennae^41^, this behaviour suggests that they are able to identify non-rewarding flowers better if they land on them instead of just hovering. This could be due to a variety of reasons: for example, they may be able to detect scent-marks with their feet (as is the case for flies and butterflies^42^, it may simply give them more time to detect any scent-marks with their antennae, or it may allow them to get closer to the scent-mark. This shows that bumblebees can’t always reject unrewarding flowers based on its associated scent-mark without landing. This was especially true when both unrewarding and rewarding flowers were scent-marked, in which case it wasn’t unusual for the bees to enter or even drink from unrewarding flowers. Furthermore, any hovering, or hovering then landing, behaviour was very brief, typically lasting for less than a second. This suggests that the amount of time required to discriminate between rewarding and unrewarding flowers using scent-marks is likely negligible.

The bees may also have expected the presence of a reward within the flower by its associated scent-mark. Thus, they were attracted to these flowers, rather than simply drinking from them by default, as the scent-mark associated with the absence of reward would not have been detected. Given that bumblebees living under laboratory conditions may become more attracted to scent-marked flowers due to repeat exposure to rewarding flowers that have not had their scent-marks removed^3^,^19^,^30^, and that they do not innately prefer flowers that have or have not been scent-marked^29^, it is likely that they are detecting the presence of a reward within a flower by its associated scent-mark.

For each experiment the bees varied in the speed in which they learnt the associations and were able to distinguish between the two flower types. This was apparent from the number of training bouts performed before testing (Fig. 4), suggesting a difference in the amount of repetition required before making the association. They required significantly more training bouts in the Experiment 2 than in Experiment 1, and their learning ability was also more variable in Experiment 2; again, evidence that they found it harder to discriminate between the two flower types when both flowers were scent-marked. Furthermore, two out of the fourteen bees used in Experiment 2 and seven of the nineteen bees used in Experiment 3 failed to progress to the testing phase, whereas all of the bees tested in Experiment 1 progressed to the testing phase. The learning speed was highly variable for the bees in Experiment 3, with some bees learning almost immediately and some requiring the maximum number of training bouts (Fig. 4). This high variability may be because each flower type was scent-marked by more than one bee and so discriminating between them was a more difficult task, thus causing many bees to fail. Furthermore, none of the bees tested in Experiment 3 would have previously encountered the scent-mark of a non-nestmate, and so this could be a reason for the few bees that learned the task relatively quickly.

Although the task itself is likely to be quite difficult, it is possible that the number of learning bouts was affected by the level of scent-mark present on the flowers. If the level of scent-mark present on the flowers marked by the bees being tested had increased over repeated visits this would have been consistent across individuals in Experiments 1 and 2. Conversely, the amount of scent-mark present on the flowers marked by the test bees in Experiment 2 should be sufficient to elicit a response due to what was observed in Experiment 1. The amount of scent-mark present on the flowers marked by nestmates in Experiment 2 should also be enough for the test bees to detect as even a single visit from a bumblebee is enough to elicit a response^20^. Furthermore, similar learning speeds have been observed in experiments involving flower colour discrimination^43^.

In Experiment 1 the bees spent much more time on the unmarked flowers (rewarding in the training phase) compared to the scent-marked flowers (unrewarding in the training phase) they landed on. This suggests that they don’t simply unlearn the association between rewarding flowers and the absence of a scent-mark; they stay longer on these flowers and repeatedly drink, albeit water, search, and lick around the well expecting to find a sucrose reward. Similarly for Experiments 2 and 3, we observed that they spent more time on flowers that were originally rewarding. However, they seemed to spend less time on these flowers compared with Experiment 1, suggesting that the learned association was stronger for Experiment 1. So not only do they find it easier to discriminate between flower types and learn faster when one of the flower types is unmarked, they also seem more certain about what they have learnt previously, even when they encounter contradictory information. This is likely due to the decreased ambiguity between scent-marked and unmarked flowers.

It is worth noting that frequently the bumblebees were initially repelled by the clean flowers (i.e. when either the bees being tested or their nestmates were required to scent-mark clean flowers), apparent by prolonged hovering behaviour without landing, which is consistent with previous experiments^3^,^19^,^30^. This is because bumblebees under laboratory conditions are not used to foraging on clean flowers that are rewarding, as flowers are often replenished with sucrose solution without having their scent-marks removed through cleaning. There was no evidence that any of the bees initially preferred one of the flowers types in Experiments 2 or 3. Although, the bees would often fly around the flight arena ignoring the flowers for prolonged periods after drinking from a flower that contained water during the testing phase, but had originally contained sucrose in the training phase (observed predominantly in Experiment 3). Foraging on unexpectedly unrewarding flowers may persuade the bees to seek alternative flower types, which is a possible reason for this observed behaviour.

To our knowledge there has been no attempt to assess a bumblebee’s ability at discriminating between these different types of scent-marks. Until now all that could be said is that bumblebees can detect and associate scent-marks from a given source with rewarding flowers^31^ or unrewarding flowers^33^. What we have found is it that not only are bumblebees able to distinguish between unmarked flowers and flowers that they themselves have scent-marked, but they can also distinguish between flowers scent-marked by themselves and flowers scent-marked by their nestmates, and flowers scent-marked by their nestmates and flowers scent-marked by non-nestmates. It shows that bumblebees have the ability to distinguish between scent-marks that are potentially very similar in their chemical composition (hydrocarbons) and they can use this ability to improve their foraging success. The degree of similarity in the composition of the scent-marks of a bumblebee, its nestmates, and its non-nestmates, can be seen in the variance in the amount of six of the main hydrocarbons found within the scent-mark of *B. terrestris*^3^,^4^, each varying differently between individuals of this species. Furthermore, the hydrocarbon composition of bumblebee scent-marks varies more between species (*B. terrestris, B. pascuorum*, and *B. lapidarius*) than within species, mainly due to some of the hydrocarbons being absent from one species scent-mark but present in another.

Showing that individuals are able to identify scent-marks left by conspecifics who are not themselves, or even their nest-mates, contributes towards our understanding of kin and nestmate recognition, which are important mechanisms in kin selection^44^. This is especially true of nestmate recognition, given that the composition of hydrocarbons found in bumblebee scent-marks and their epicuticular hydrocarbons are almost identical^25^ and that this hydrocarbon composition varies between bumblebee species^4^.

Studies like this one could be performed with other important pollinating insects that are also able to detect and use scent-marks, which would help to understand and compare the foraging behaviour of the main insect pollinators. For example, sweat bees are able to use scent-marks deposited by conspecifics^45^ and heterospecifics^46^. Stingless bees use scent-marks deposited by conspecifics whilst foraging^47^ and their behaviour can also depend on the species of stingless bee that deposited them, which can be quite aggressive^48^.

Honeybees are able to use scent-marks as social cues during foraging^33^,^34^,^49^,^50^. Furthermore, when using scent-marks as an indication of depleted flowers and therefore rejecting these flowers, honeybees show a different level of response towards flowers containing their own scent-mark than towards flowers containing their nestmate’s scent-mark^51^. Although in this study the bees are rejecting all flowers containing a scent-mark, the findings suggest that honeybees may be able to discriminate between their own scent-mark and the scent-mark of their nestmate. There is also evidence to suggest that honeybees are able to discriminate between the scent-marks of conspecifics and heterospecifics (bumblebees), which influences flower rejection^52^. In addition, although solitary bees have been found to have inferior learning capabilities than social bees^43^, they can still distinguish between visited and unvisited flowers using scent-marks deposited by conspecifics^53^ or by heterospecifics^21^. Furthermore, solitary bees (*Anthophora plumipes*) have been found to respond differently towards flowers visited by themselves and flowers visited by a conspecific^54^. It is suggested that this ability may help solitary bees compete with conspecifics for floral resources. To our knowledge, there is no suggestion of this ability in bumblebees, which makes our finding that bumblebees are able to discriminate between their own scent-mark and that of their nestmates all the more important.

Our findings could help shape future experimental or theoretical studies involving a bumblebee’s foraging behaviour, especially when being considered alongside their nestmates or multiple nests. It would be interesting to apply these findings to larger-scale, field-based experiments to discover the effect of varying the number of foragers within a nest, or having multiple nests with overlapping foraging ranges, on individual bumblebee foraging behaviour. Furthermore, behaviours such as traplining^55^, where a forager visits resources in a specific order, may be affected by the presence of scent-marks^56^, and this effect may vary depending on the source of the scent-mark^48^. Thus, gathering information on a pollinator’s ability to use and discriminate between scent-marks would be beneficial for studies that involve traplining behaviour^57^, especially when more than one pollinator is being considered^58^.

Finally, responding to scent-marks is an important, albeit indirect, form of animal interaction and quantifying its effects would inform pollinator foraging models^59^. This sort of interaction is termed stigmergy^60^,^61^, whereby individuals respond to each other through an indirect modification of the environment, in our case the information of past presence of a bumblebee on a flower. There are examples of stigmergic models that have focused on studying a population’s spatiotemporal patterns^62^,^63^ as well as animal nest architecture^64^,^65^. Although there are examples of stigmergy to aid colony level foraging, for example in the form of pheromone trails^66^, we feel that there is still an opportunity to further develop stigmergic models in the foraging context. Given our empirical findings we hope to stimulate the development of collective foraging models to identify relative efficiencies of solitary versus eusocial foragers, such as bumblebees.

## Materials and Methods

### Bumblebees and set-up

Buff-tailed bumblebees *Bombus (Bombus) terrestris* subsp. *audax* (Harris, 1776) were supplied by Agralan (Swindon, UK), which were used in all experiments, and by Fargro (Arundel, UK), which were used only for Experiment 3. They were housed in cardboard nestboxes in a room lit with natural light mimicking lightbulbs, at a temperature of around 20 °C, and humidity of around 40%. Each nest box was attached to a plywood flight arena (112 × 75 × 30 cm) with a UV-penetrable Plexiglas^®^ lid and green tape covering the floor. The bees could travel between their nestbox and flight arena *via* a transparent plastic tube (30 × 1.5 cm ø), with their access controlled using removable stoppers along the tube. The bees were allowed to acclimatise for at least one week before any experimentation took place. Each bee, identifiable by different coloured paint marks, took around six hours to complete the experiment, which was divided into a training phase and a testing phase (described below). The bees drank from flowers that were made artificially and were identical in appearance (Fig. 1A). The flowers were created using sterile transparent cylindrical plastic pots with a white lid (10 cm × 4 cm ø, 60 ml – Sterilin™, Fisher Scientific UK, Loughborough, UK) and an opaque well for storing a reward or water. The well was covered with a translucent cylindrical cover (made using the end of a plastic pipette – see Fig. 1A), that forced the bee to walk over a fixed area of the flower before it was able to assess the contents of the well. This ensured that each bee entered the flower in the same way; being guided to the same patch of flower before landing and drinking increased the likelihood that each bee was exposed to any scent-mark that may have been deposited onto the flower. A semi-circular piece of filter paper (No. 1 qualitative circles, 42.5 mm ø – Whatman, GE Healthcare, Little Chalfont, UK) was attached to the entrance of the flower (Fig. 1A); the surface of the filter paper was more textured and absorbent than the plastic lid and was added to increase the amount of scent-mark deposited onto the flower by the bees. Latex gloves were worn at all times and cleaned using ethanol before and after touching any of the floral components to minimise the transfer of scents. The flowers were also cleaned with ethanol before being used in the experiments.

### Experiment 1: Own scent-mark vs No scent-mark

Twelve bees were tested from three nests (four from each). Firstly, each bee was initially exposed to two unmarked flowers each containing two drops of sucrose solution (30% v/v). Two drops of water were then added to these two visited flowers after ensuring there was no excess sucrose solution. These were then used as the scent-marked flowers for the duration of the experiment with that bee, along with two unmarked flowers that contained two drops of sucrose solution (Table 1). Each bee first went through a training phase consisting of multiple training bouts, followed by a testing phase – consisting of both testing bouts and training bouts – to discount for sucrose detection. The only difference between the training phase and the testing phase was that the rewarding flower type in the training phase (see Table 1) contained water in the testing phase. A bout was defined as the bee entering the flight arena, interacting with at least one flower, and then exiting the flight arena. Forms of interaction with the flower are defined as follows: the bee hovers facing the flower but does not land (Hover), lands on the flower (Land), enters the flower but does not touch the well with its proboscis or antennae (Enter), and drinks from the well or extends proboscis (Drink).

For the training phase, the four flowers were arranged in either a square or a diamond formation (Fig. 1B), which was alternated for each training bout, and the scent-marked and non-scent-marked flowers were positioned randomly within each arrangement. After each training bout any of the initially unmarked flowers that the bee landed on were replaced with new unmarked ones. The initially scent-marked flowers remained in the flight arena, and were not cleaned, regardless of whether the bee had visited them or not during the bout. The training phase was continued for at least five training bouts (not including the initial exposure to two unmarked flowers) and until the bee interacted with, but did not drink from, at least one scent-marked flower and drank from at least one non-scent-marked flower for three consecutive training bouts. After this criterion was met the bee progressed to the testing phase. All twelve bees progressed to the testing phase.

The testing phase consisted of three testing bouts each separated by two training bouts. During each testing bout all four flowers contained water and so were unrewarding. For each testing bout the bee was exposed to four flowers: two of these were scent-marked by the bee (those used in the training phase) and two were not. Consistent with the training phase, the flowers were distributed in one of the two alternating formations with the flower types being randomly positioned. The training bouts involved the same set-up as the training phase: two scent-marked flowers containing water and two unmarked flowers containing sucrose solution. We recorded the numbers of each type of interaction exhibited by each bee for each initial flower visit during each of the three testing bouts, therefore twelve (six from scent-marked flowers and six from non-scent-marked flowers) occurrences of behaviour for each bee were obtained. A single interaction with a flower could involve one or more of the behaviours mentioned previously. Each testing bout was complete when the bee interacted with each of the four flowers or exited the flight arena. Only the bee’s initial interaction with each flower was recorded. If the bee did not interact with a flower then this was recorded as no interaction.

### Experiment 2: Own scent-mark vs Nestmates’ scent-mark

Fourteen bees from four nests undertook the experiment, with no more than four bees from the same nest. Two of the bees initially trained did not qualify for the testing, so test data were collected for twelve bees. In this experiment the bees were being trained to discriminate between flowers scent-marked by themselves and flowers scent-marked by other nestmates (Table 1).

For the training phase, the two flowers scent-marked by the test bee were obtained in the same way as they were in Experiment 1. During training, these again contained water. Two other flowers were scent-marked by the test bee’s nestmates before each training bout and contained two drops of sucrose solution. To obtain these, two bees (not including the test bee) were allowed into the flight arena while the test bee was inside the nest; the flight arena contained two unmarked flowers each containing two drops of sucrose solution. Once each bee had landed on both flowers, removed the sucrose solution, and returned back to the nest, these two flowers were replenished with two drops of sucrose solution. The rest of the training phase and the criterion for progressing to the testing phase was identical to that of Experiment 1.

The testing phase was the same as for Experiment 1 (three testing bouts each separated by two training bouts), apart from two of the flowers were now scent-marked by the test bee’s nestmates instead of being unmarked. Again, for the testing bouts all four flowers contained two drops of water and the training bouts involved the same set-up as the training phase.

### Experiment 3: Non-Nestmates’ scent-mark vs Nestmates’ scent-mark

Nineteen bees from four nests were initially trained, of which twelve reached test criteria. No more than six bees from the same nest undertook the training and no more than four bees from the same nest were tested. In this experiment the bees were being trained to discriminate between flowers scent-marked by non-nestmates and flowers scent-marked by nestmates (Table 1). We required two flowers scent-marked by bees within the same nest as the test bee and two flowers scent-marked by bees that were members of a different nest. The different nests were obtained from different suppliers (Agralan and Fargro), which were of the same subspecies (*audax*). Different suppliers were used to reduce the relatedness of the two nests, as those from the same supplier may be more closely related. A pair of bees were temporarily removed from the test bee’s nest, and another pair were removed from a different nest from a different supplier. Each pair were housed separately underneath clean upturned petri dishes. Two semicircles of filter paper were placed underneath each of the petri dishes to allow the bees to walk over them. The two pieces of filter paper from the petri dish housing the test bee’s nestmates were attached to two clean flowers containing two drops of sucrose. The two pieces of filter paper from petri dish housing the non-nestmates were attached to two clean flowers containing two drops of water. The rest of the training phase and the criterion for progressing to the testing phase was identical to that of Experiment 1.

The testing phase was the same as for Experiment 2 (three testing bouts each separated by two training bouts), apart from two of the flowers were now scent-marked by non-nestmates instead of by the test bee. For the testing bouts, all four flowers contained two drops of water. The training bouts involved the same set-up as the training phase.

### Data analysis

We treated each bee as a single data-point as they are unique individuals; we did not take colony into consideration as there are other factors that would influence a bee’s foraging behaviour more than the colony it belongs to^67^. For each experiment, the occurrence of behaviours each individual showed towards each flower type were compared using a pairwise Mann-Whitney-Wilcoxon test. We also analysed the difference in how the bees behaved towards the two flower types depending on their previous behaviour, again using a pairwise Mann-Whitney-Wilcoxon test. For example, the occurrence of landing given that the bee hovered first. We only considered hovering and landing as the dependent behaviours here as there were a sufficient number of occurrences of these behaviours in each experiment. For a subset (n = 5) of bees from Experiment 1 we recorded the length of time they each spent on the flowers they landed on (taken to be from when the bee landed on the flower to when it departed) during the three separate testing bouts. Due to the small sample size we used a paired *t*-test^68^ to compare the difference in this mean duration for the two flower types. The number training phase foraging bouts required before progressing to the testing phase was compared between Experiments 1 and 2 using a Kolmogorov-Smirnov test. In Experiment 3 scent-marks were obtained using a slightly different method, so direct comparison with Experiments 1 and 2 was avoided. Any data represented as a boxplot includes the median, lower and upper quartile, and outliers; outliers are defined as being more than one interquartile range from the box, with the whiskers extending to the most extreme data-point within this.

### Ethical note

Outside of the experimental period the nest had standard *ad libitum* access to all the resources known to be required to maintain standard laboratory behaviour. After experiments had finished the Bumblebees were allowed to forage under fully stocked *ad libitum* conditions until the colony was not producing foragers, after which they were euthanised by placing the nest box in the freezer. There is no requirement by the University of Bristol to seek ethical approval for experiments with insects, and the sample sizes were designed to minimise the number of individuals used in the experiment.

## Additional Information

**How to cite this article**: Pearce, R. F. *et al*. Bumblebees can discriminate between scent-marks deposited by conspecifics. *Sci. Rep.* **7**, 43872; doi: 10.1038/srep43872 (2017).

**Publisher's note:** Springer Nature remains neutral with regard to jurisdictional claims in published maps and institutional affiliations.

## Supplementary Material

Supplementary Dataset 1

Supplementary Dataset 2

## Figures and Tables

**Table 1:** The different scent-mark treatments for all learning bouts of each of the three experiments.

Experiment	Unrewarding flowers: contain water	Rewarding flowers: contain sucrose solution
1	Own	Absent (unmarked)
2	Own	Nestmates
3	Non-nestmates	Nestmates
