# Supplementary material for: Bumblebees can discriminate between scent-marks deposited by conspecifics
Source: Sci Rep. 2017 Mar 7;7:43872. doi: 10.1038/srep43872 (PMC5339730; doi:10.1038/srep43872)
Supplement: Supplementary Dataset 2 [file srep43872-s2.pdf]

Bumblebees can discriminate between scent-marks deposited by conspecifics

Richard F. Pearce[*] [a, b], Luca Giuggioli [b, c], Sean A. Rands [a]

[a] School of Biological Sciences, University of Bristol, Bristol, UK

[b] Bristol Centre for Complexity Sciences, University of Bristol, Bristol, UK

[c] Department of Engineering Mathematics, University of Bristol, UK

[*] Corresponding author: Richard.Pearce@Bristol.ac.uk

School of Biological Sciences, Life Sciences Building, University of Bristol,

Tyndall Avenue, Bristol BS8 1TQ, UK

Description of supplementary dataset:

Under the heading **Fig 2** contains, for each of the three experiments, the number of occurrences (out of 6) of each of the foraging behaviours towards each of the two flower types (different scent-marks) for each of the 12 bumblebees tested.

Under the heading **Fig 3** contains, for each of the three experiments, the proportion of each dependent foraging behaviour towards each of the two flower types (different scent-marks) for each of the 12 bumblebees tested.

Under the heading **Fig 4** contains, for each of the three experiments, the number of training bout required (learning phase) before progressing onto the testing phase for each of the 12 bumblebees tested.

Under the heading **Durations** contains, for each of the three experiments, the duration (seconds) of initial flower visits to each of the two flower types (different scent-marks) for the subset of bumblebees tested.
